# 阿伐替尼桥接异基因造血干细胞移植治疗伴有KIT突变的复发/难治性RUNX1-RUNX1T1阳性急性髓系白血病7例疗效分析

**DOI:** 10.3760/cma.j.cn121090-20240526-00188

**Published:** 2024-08

**Authors:** 思懿 韩, 海侠 周, 悦 韩, 德沛 吴

**Affiliations:** 苏州大学第一附属医院，苏州 215000 The First Affiliated Hospital of Soochow University, Suzhou 215000, China

**Keywords:** 阿伐替尼, 异基因造血干细胞移植, 急性髓系白血病, 融合基因，RUNX1-RUNX1T1, Avastinib, Allogeneic hematopoietic stem cell transplantation, Acute myeloid leukemia, Fusion gene, RUNX1-RUNX1T1

## Abstract

**目的:**

探讨阿伐替尼桥接异基因造血干细胞移植（allo-HSCT）治疗伴KIT突变复发/难治性RUNX1-RUNX1T1阳性急性髓系白血病（AML）的疗效。

**方法:**

对2019年6月至2023年6月期间在苏州大学附属第一医院接受阿伐替尼桥接allo-HSCT治疗的7例伴RUNX1-RUNX1T1融合基因与KIT突变的复发/难治性AML患者进行回顾性研究。

**结果:**

7例AML患者中男1例，女6例，中位年龄37（18～56）岁。7例患者均伴有KIT突变（D816V突变阳性5例，D816Y突变阳性2例）。难治患者2例，复发患者5例（均为骨髓复发）。所有患者移植前至少完成1个疗程阿伐替尼治疗。4例患者在阿伐替尼治疗后获得完全缓解（CR），6例患者KIT基因突变转阴，1例患者KIT基因突变负荷较前下降。全相合无关供者移植3例，单倍体移植4例。所有患者均接受改良Bu/Cy预处理方案。移植后所有患者均顺利植入，复查骨髓象均达CR且微小残留病（MRD）转阴，5例患者融合基因转阴。移植后2例患者死于感染。

**结论:**

阿伐替尼桥接allo-HSCT对于伴有KIT-D816突变的RUNX1-RUNX1T1阳性AML患者可能是一种有效、安全的治疗新策略。

## Abstract (en)

**Objective:**

To evaluate the efficacy of avatinib plus allogeneic hematopoietic stem cell transplantation（allo-HSCT）for the treatment of recurrent/refractory RUNX1-RUNX1T1 positive acute myeloid leukemia（AML）with KIT mutations.

**Methods:**

A retrospective study was conducted on the clinical data of seven relapsed/refractory AML patients containing the RUNX1-RUNX1T1 fusion gene and KIT mutation who received afatinib plus allo-HSCT treatment at the First Affiliated Hospital of Soochow University from June 2019 to June 2023.

**Results:**

The seven AML patients included one male and six females with a median age of 37（18–56）years. All seven patients had KIT mutations（five positive for D816V and two positive for D816Y）. There were two refractory patients and five relapsed patients（all of whom had bone marrow recurrence）. All patients had to complete at least one course of treatment with afatinib before transplantation. Four patients achieved complete remission（CR）after treatment with afatinib, six patients had negative KIT gene mutations, and one had a decreased KIT gene mutational burden. There were three cases of unrelated identical transplantation and four cases of haploidentical transplantation. All patients received the modified Bu/Cy pretreatment regimen. After transplantation, all patients were successfully implanted and a bone marrow examination showed CR and minimal residual disease turned negative. Five patients exhibited negative fusion genes. Two patients died from infection following transplantation.

**Conclusion:**

Afatinib plus allo-HSCT may be an effective and safe new treatment strategy for RUNX1-RUNX1T1 positive AML patients with KIT-D816 mutation.

t（8;21）（q22;q22）是急性髓系白血病（AML）患者中常见的染色体异常，会产生RUNX1-RUNX1T1（AML1-ETO）融合基因[1]，进而导致异常融合蛋白并阻碍髓系分化[2]–[3]。根据2022年欧洲白血病网（ELN）遗传风险分层，RUNX1-RUNX1T1融合基因阳性AML属于低风险组，尽管如此，这些患者中仍有约40％的复发率[4]–[5]。以往研究证明，KIT-D816基因突变是RUNX1-RUNX1T1融合基因阳性AML患者的独立不良预后因素，具有更高的复发率和死亡率[6]–[9]，此外，常规化疗对伴有KIT突变的RUNX1-RUNX1T1阳性AML患者的完全缓解（CR）率仅为11％～19％，中位生存时间≤6个月[10]–[12]。这些结果凸显了伴有KIT突变的复发/难治性RUNX1-RUNX1T1阳性AML患者缺乏高效、应答率高的治疗方案。《中国复发难治性急性髓系白血病诊疗指南（2023年版）》建议的治疗手段包括靶向治疗或化疗后序贯异基因造血干细胞移植（allo-HSCT）[13]，阿伐替尼在AML患者中使用相对较少，近年来我中心采用阿伐替尼桥接allo-HSCT治疗7例伴有KIT-D816突变的RUNX1-RUNX1T1阳性AML患者，获得较好疗效。

## 病例与方法

一、病例资料

本项回顾性研究纳入2019年6月至2023年6月期间在苏州大学附属第一医院接受阿伐替尼桥接allo-HSCT治疗的7例伴有RUNX1-RUNX1T1融合基因与KIT突变的复发/难治成人AML患者。

二、阿伐替尼用药方案

所有患者移植前至少完成1个疗程阿伐替尼治疗，具体用药方案：100～200 mg/d×7～14 d，根据患者血象及具体情况调整。其中1例患者因KIT突变持续未转阴但患者KIT突变呈下降趋势，为进一步降低KIT突变负荷，将阿伐替尼疗程延长至28 d。

三、观察指标

一般基线资料包括患者的年龄、性别、病程、阿伐替尼使用前缓解情况；疗效指标包括使用阿伐替尼前后血常规、肝肾功能、骨髓细胞形态学、微小残留病（MRD）变化及RUNX1-RUNX1T1融合基因水平等。

四、定义

RUNX1-RUNX1T1阳性AML缓解：骨髓原始细胞<5％，外周血中无原始细胞。参照《中国复发难治性急性髓系白血病诊疗指南（2023年版）》，骨髓复发：AML CR后外周血再次出现白血病细胞或骨髓中原始细胞≥5％（除外巩固化疗后骨髓再生等其他原因）或髓外出现白血病细胞浸润。难治：①经过标准方案治疗2个疗程无效的初治病例；②CR后经过巩固强化治疗，12个月内复发者；③在12个月后复发但经过常规化疗无效者；④2次或多次复发者；⑤髓外白血病持续存在者。MRD复发指MRD阴性（<10^−3^水平的肿瘤细胞）后流式细胞术再次检测到大于10^−3^（0.1％）水平的肿瘤细胞（骨髓达CR）或融合基因转为阳性。分子学复发指融合基因转为阳性。急性移植物抗宿主病（GVHD）诊断标准参照文献[14]。慢性GVHD诊断标准参照文献[15]。中性粒细胞植入标准：中性粒细胞绝对计数（ANC）≥0.5×10^9^/L连续3 d；血小板植入标准：PLT≥20×10^9^/L连续7 d且脱离血小板输注。EB病毒（EBV）定量测定EBV-DNA≥1×10^2^拷贝/ml诊断为EBV感染，巨细胞病毒（CMV）定量测定CMV-DNA≥1×10^2^拷贝/ml诊断为CMV感染。

五、不良反应判定

使用阿伐替尼的不良反应中血液学毒性及非血液学毒性均依据CTCAE 5.0标准。

六、随访

通过查阅门诊/住院病历和电话随访获得患者生存资料。

## 结果

一、患者一般临床资料

7例复发/难治性AML患者中男1例，女6例。难治患者2例，复发患者5例，均为骨髓复发（表1）。所有患者均接受改良Bu/Cy预处理方案：白消安3.2 mg·kg^−1^·d^−1^×3 d，环磷酰胺50 mg·kg^−1^·d^−1^×2 d，兔抗人胸腺细胞免疫球蛋白2.5 mg·kg^−1^·d^−1^×4 d，详见表2。

**表1:** 7例伴有KIT突变的复发/难治性RUNX1-RUNX1T1阳性急性髓系白血病患者的临床特征及阿伐替尼使用情况和疗效

例号	性别	年龄（岁）	治疗时疾病状态	阿伐替尼剂量及疗程	阿伐替尼使用前骨髓原幼细胞、MRD及融合基因表达
1	女	40	骨髓复发	200 mg/d×14 d	骨髓原幼细胞15.5%，MRD 0.43%，RUNX1-RUNX1T1定量584.21%
2	女	37	骨髓复发	200 mg/d×14 d	骨髓原幼细胞8%，MRD 1.7%，RUNX1-RUNX1T1定量56.99%
3	女	54	骨髓复发	①100 mg/d、第1~5天，200 mg/d、第6~12天； ②200 mg/d×28 d	①骨髓原幼细胞35%，MRD 53.59%，RUNX1-RUNX1T1定量649.68%； ②骨髓原幼细胞2%，MRD 0.18%，RUNX1-RUNX1T1定量4.51%
4	女	27	骨髓复发	100 mg/d×14 d	骨髓原幼细胞59.5%，MRD 42.46%，RUNX1-RUNX1T1定量250.46%
5	女	18	骨髓复发	200 mg/d×7 d	骨髓原幼细胞79%，MRD 34.4%，RUNX1-RUNX1T1定量1260.19%
6	女	56	骨髓复发	200 mg/d×7 d	骨髓原幼细胞60%，MRD 56.62%，RUNX1-RUNX1T1定量330.43%
7	男	36	骨髓复发	200 mg/d×14 d	骨髓原幼细胞25%，MRD 25.8%，RUNX1-RUNX1T1定量314.6%

**Table:** 

例号	阿伐替尼使用前KIT基因突变	其他共突变情况	阿伐替尼使用后骨髓原幼细胞、MRD及融合基因表达	阿伐替尼使用后KIT基因突变
1	KIT D816Y 4.3%	ASXL2、SF3B1、SMC1A	骨髓原幼细胞14%，MRD 0.45%，RUNX1-RUNX1T1定量60.06%	阴性
2	KIT D816V 26.3%	BCOR、BRCA2	骨髓原幼细胞未见，MRD阴性，RUNX1-RUNX1T1定量4.01%	阴性
3	①KIT D816V 38.31%； ②KIT D816V 17.9%	无	①骨髓原幼细胞2%，MRD 0.18%，RUNX1-RUNX1T1定量4.51%； ②骨髓原幼细胞未见，MRD阴性，RUNX1-RUNX1T1定量0.04%	①KIT D816V 15.83% ②阴性
4	KIT D816V 45.4%	CSMD1、SETD2	骨髓原幼细胞78%，MRD 72.3%，RUNX1-RUNX1T1定量829.98%	KIT D816V 77.8%
5	KIT D816V 63.8%	DHX15	骨髓原幼细胞29%，MRD 4.76%，RUNX1-RUNX1T1定量667.47%	阴性
6	KIT D816V 14.4%	FLT3-ITD、TET2、DNMT3A	骨髓原幼细胞未见，MRD阴性，RUNX1-RUNX1T1定量阴性	阴性
7	KIT D816Y 48.1%	无	骨髓原幼细胞0.5%，MRD 0.1%，RUNX1-RUNX1T1定量4.06%	阴性

**注** MRD：微小残留病

**表2:** 7例伴有KIT突变的复发/难治性RUNX1-RUNX1T1阳性急性髓系白血病患者阿伐替尼治疗后异基因造血干细胞移植资料及疗效

例号	移植前疾病状态	供者	移植后患者骨髓原幼细胞、MRD及融合基因表达	移植后患者KIT基因突变	嵌合度（％）	GVHD	EBV感染	CMV感染	生存期（d）
1	NR	全相合无关	骨髓原幼细胞未见，MRD阴性，RUNX1-RUNX1T1定量阴性	阴性	98.00	肠道急性GVHD，皮肤慢性GVHD	阴性	阳性	536
2	CR	单倍体	骨髓原幼细胞未见，MRD阴性，RUNX1-RUNX1T1定量阴性	阴性	99.10	无	阴性	阴性	632
3	CR	单倍体	骨髓原幼细胞未见，MRD阴性，RUNX1-RUNX1T1定量0.15％	阴性	98.30	皮肤慢性GVHD	阴性	阳性	305（死于感染）
4	NR	全相合无关	骨髓原幼细胞未见，MRD阴性，RUNX1-RUNX1T1定量阴性	阴性	98.80	无	阴性	阴性	527
5	NR	单倍体	骨髓原幼细胞未见，MRD阴性，RUNX1-RUNX1T1定量阴性	阴性	99.00	无	阴性	阳性	486
6	CR	全相合无关	骨髓原幼细胞未见，MRD阴性，RUNX1-RUNX1T1定量阴性	阴性	99.00	皮肤慢性GVHD，眼部慢性GVHD	阳性	阴性	775
7	CR	单倍体	骨髓原幼细胞未见，MRD阴性，RUNX1-RUNX1T1定量15.86％	阴性	98.20	皮肤慢性GVHD	阴性	阳性	296（死于感染）

**注** MRD：微小残留病；GVHD：移植物抗宿主病；EBV：EB病毒；CMV：巨细胞病毒；CR：完全缓解；NR：未缓解

二、疗效

7例患者中有2例死亡，死亡原因均为感染，其余5例仍存活。4例在使用阿伐替尼后达CR，其中，2例MRD为阴性，2例MRD为阳性（包括融合基因阳性），另外3例在使用阿伐替尼后仍处于NR状态，但骨髓原幼细胞、MRD及融合基因负载量较使用前均有下降。7例患者均伴有KIT突变，其中D816V突变阳性5例，D816Y突变阳性2例。全部7例患者使用阿伐替尼的疗效见表1。阿伐替尼治疗后，6例患者KIT基因突变转阴，另外1例KIT基因突变负荷较前下降。所有患者在阿伐替尼治疗后桥接allo-HSCT，其中全相合无关供者移植3例，单倍体移植4例。移植后所有患者均顺利植入，短串联重复序列嵌合度>95％，复查骨髓象均达CR且MRD均转阴，5例患者融合基因转阴。移植后发生EBV感染1例，CMV感染4例，皮肤慢性GVHD 4例，肠道急性GVHD 1例，眼部慢性GVHD 1例。详见表2。

三、不良反应

1. 非血液学不良反应：7例患者中2例出现皮疹，1例出现胆红素升高，1例出现头痛，1例出现呕吐，1例出现面部水肿。

2. 血液学不良反应：7例患者中1例发生Ⅲ级中性粒细胞减少，3例发生Ⅲ/Ⅳ级血红蛋白减少，2例发生Ⅲ/Ⅳ级血小板减少。

## 讨论

t（8；21）（q22；q22）是AML患者中最常见的染色体易位，占成年AML的5％～8％，根据2022年ELN遗传风险分层，t（8，21）/RUNX1-RUNX1T1阳性AML属于低危组，仍有相当大比例的患者经历复发甚至死亡[4]–[5]。尽管近年AML的治疗方式逐渐增加，但复发/难治性AML的治疗仍然是我们当前临床工作中的巨大挑战。复发/难治性AML异质性强，预后极差，5年生存率仅为10％[16]–[17]。近年来，随着靶向药物疗效的肯定，越来越多的靶向药物被应用于临床，《中国复发难治性急性髓系白血病诊疗指南（2023年版）》建议的治疗手段包括靶向治疗或化疗后序贯allo-HSCT，因此，靶向治疗成为近年来AML领域的热点，为一些复发/难治性AML患者带来再次缓解的机会[13]。FLT3-ITD和KIT-D816热点突变是RUNX1-RUNX1T1阳性AML患者不良预后相关的独立危险因素。随着索拉非尼、吉非替尼、米哚妥林等药物的上市，FLT3-ITD突变患者的生存率有所提高，但这些药物在KIT-D816突变患者中的疗效并不显著[18]。尽管造血干细胞移植能显著提高复发/难治性AML患者生存率，但患者移植时的疾病状态是影响其移植后预后的重要因素，对复发/难治性AML患者来说，在移植前进一步降低肿瘤负荷，尽可能达到缓解至关重要，阿伐替尼是一种高选择性KIT-D816抑制剂，其对KIT-D816突变的选择性较米哚妥林更高，体外药效也高出10倍[19]–[20]，国外已批准阿伐替尼用于侵袭性肥大细胞增多症[21]。此外，阿伐替尼具有较强的渗透能力，对髓外浸润患者也有一定的治疗效果[22]–[24]，可使伴有KIT-D816突变患者中的获得完全、持久的临床反应和分子缓解，这些都为阿伐替尼在伴有KIT-D816突变的患者应用提供了有力依据。本组7例患者的回顾性研究结果显示选择阿伐替尼作为移植前桥接方案有显著的治疗效果，可在一定程度上延长患者移植后生存时间。除此之外，与常规治疗相比，接受阿伐替尼治疗的患者可以获得达到更深度的缓解，7例患者中有6例在接受阿伐替尼治疗后KIT突变基因转阴，且在移植后持续保持阴性。

Kong等[25]应用阿伐替尼治疗20例allo-HSCT后对免疫疗法无效的伴KIT突变RUNX1-RUNX1T1阳性AML患者，其中9例患者的RUNX1-RUNX1T1转录物转为阴性。结合本中心情况，阿伐替尼治疗难治/复发RUNX1-RUNX1T1阳性AML的疗效比较理想。

阿伐替尼最常见的非血液学不良事件为外周/眶周水肿、腹泻、恶心，血液学不良反应包括中性粒细胞减少、贫血和血小板减少[26]–[27]。接受阿伐替尼治疗的患者进行性血细胞减少可能不仅反映了药物相关的骨髓抑制，可能也反应了患者病灶的持续存在。血液学不良反应方面，本组7例患者中1例发生Ⅲ级中性粒细胞减少，2例发生Ⅲ/Ⅳ级血小板减少，这与国外报道阿伐替尼在侵袭性肥大细胞增多症的研究数据大致相同。3例发生Ⅲ/Ⅳ级贫血，此数据较之前的临床研究稍高，且主要发生在使用阿伐替尼靶向治疗后未缓解患者，考虑与疾病进展、高肿瘤负荷抑制骨髓造血相关。非血液学不良反应中，本组7例患者中2例出现皮疹，1例患者出现胆红素升高，1例患者出现头痛，1例患者出现呕吐，1例患者出现水肿。值得注意的是，本组7例患者移植后5例出现CMV/EBV阳性，提示在阿伐替尼桥接allo-HSCT治疗伴有KIT-D816突变的RUNX1-RUNX1T1阳性AML患者时，应更注意预防性抗病毒治疗。

总之，本研究结果显示阿伐替尼桥接allo-HSCT对于伴有KIT-D816突变的RUNX1-RUNX1T1阳性AML患者是一种有效、安全、应答性更强的治疗新策略。上述结论尚需进一步验证。
